# Chip-Scale Optomechanical Frequency Comb with a 1–70 GHz Span

**DOI:** 10.1021/acs.nanolett.5c04458

**Published:** 2025-12-12

**Authors:** Xirui Gou, William Privratsky, Wenhan Sun, Yuncong Liu, Hamed Abiri, Qing Li

**Affiliations:** ‡ Department of Electrical and Computer Engineering, 6612 Carnegie Mellon University, Pittsburgh, Pennsylvania 15213, United States; § Department of Electrical and Computer Engineering, 3463 University of Florida, Gainesville, Florida 32611, United States; ¶ School of Electrical and Computer Engineering, 1372 Georgia Institute of Technology, Atlanta, Georgia 30332, United States

**Keywords:** silicon carbide, optomechanical frequency comb, microdisk, optomechanical oscillator

## Abstract

An optomechanical frequency comb arises from the nonlinear interaction between optical and mechanical modes in a cavity, with its repetition rate set by the mechanical frequency. Despite promising applications in the microwave domain, previous demonstrations have been limited in spectral range due to inherently low mechanical frequencies. Here, we report an optomechanical comb with a record modulation span from 1 to 70 GHz, achieved by harnessing the strong optomechanical nonlinearity of a 2.5-μm-radius silicon carbide microdisk. With just 1 mW of dropped optical power, radiation pressure from a continuous-wave pump drives strong phonon lasing, generating 42 phase-locked harmonics with 1.655 GHz spacing. The combination of such broad bandwidth, low phase noise (−132 dBc/Hz at 1 MHz offset frequency), and frequency stability (<10^–7^ at 1 s of averaging time) positions this ultracompact optomechanical comb as a powerful platform for diverse applications.

## Graphical abstract

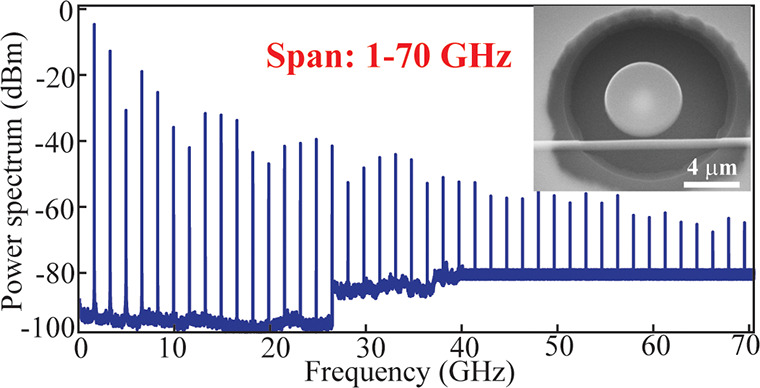

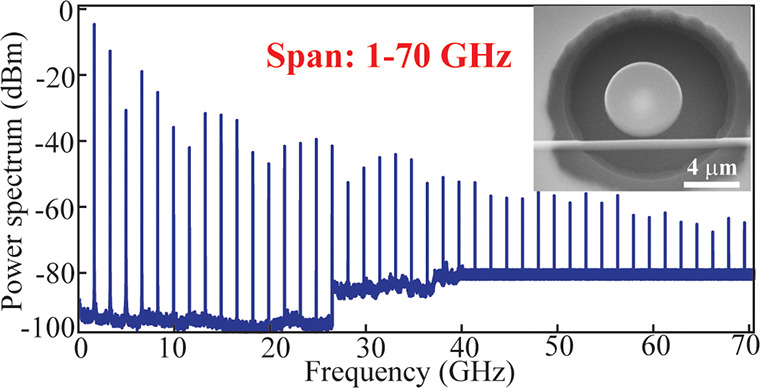

A frequency comb is a coherent source made up of equally spaced components in the frequency domain. Its distinctive feature, i.e., the maintenance of phase locking among the components, makes it a powerful tool for applications across science and technology. Optical frequency combs, for instance, have already transformed fields such as optical communications,^1^ precision metrology,^2^ imaging,^3^ sensing,^4^ microwave photonics,^5–7^ and quantum information.^8^ Much of this progress in the past decade has been driven by the microcomb technology, which leverages optical nonlinearity in low-loss microresonators to significantly reduce device size, weight, and power consumption. In contrast, phononic frequency combs (also known as mechanical frequency combs),^9^ which serve as the mechanical analog of optical frequency combs, remain less developed, particularly in terms of demonstrated spectral coverage and phase stability across the spectrum.^10–13^

A hybrid version between optical and mechanical frequency combs is an optomechanical comb, where an optical field excites mechanical motion via radiation pressure or the photoelastic effect, and the resulting optomechanical interaction gives rise to a comb spectrum that reflects signatures of both optical and mechanical domains.^14^ To date, most reported optomechanical combs employ mechanical modes with fundamental frequencies well below the gigahertz range,^15–20^ which significantly constrains their utility in microwave-frequency applications. Conversely, although high-frequency (>1 GHz) optomechanical resonators have been demonstrated across several material platforms, their relatively large optical or mechanical losses have generally precluded efficient phonon lasing and broadband comb formation.^21–23^

In this work, we demonstrate an overtone optomechanical comb in a compact silicon carbide (SiC) optomechanical resonator with an integrated optical waveguide (Figure 1). Compared to more mature materials such as silicon, SiC exhibits lower intrinsic mechanical loss, especially at cryogenic temperatures or for high-frequency mechanical modes.^24^ The device, shown in Figure 1a, is a 2.5-μm-radius microdisk fabricated on a 4H-SiC-on-insulator (4H-SiCOI) chip consisting of a 600 nm 4H-SiC layer on 2-μm silicon dioxide.^25^ The pattern is defined by electron-beam lithography (EBL) and transferred to the SiC layer using CHF_3_/O_2_ plasma etching. A second EBL step opens an undercut window, followed by wet oxide etching to release the disk.^24^ Interestingly, the mechanical quality factor (*Q*_m_) of the 2.5-μm-radius disk exhibits a local maximum at an undercut ratio (undercut width normalized by the radius) around 55–60% (Figure 1b), while further undercutting degrades *Q*_m_ until the undercut ratio is above 90%.^26^ This behavior is qualitatively different than what is reported in the literature, where a large undercut ratio is typically required for obtaining high *Q*_m_ values.^22–24^ A detailed study of anchor-loss behavior in such small 4H-SiC microdisks is left for future work.

**Figure 1:**
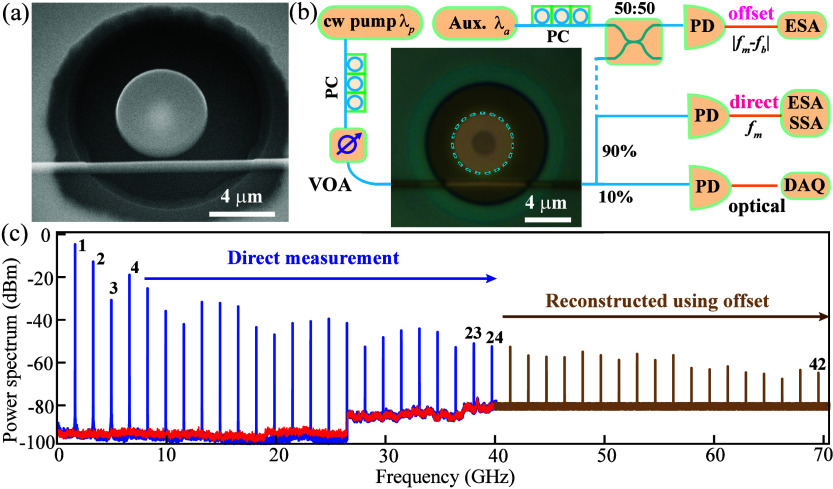
(a) Scanning electron micrograph of an undercut 2.5-μm-radius SiC microdisk. (b) Experimental schematic. PC: polarization controller. VOA: variable optical attenuator. PD: photodetector. DAQ: data acquisition card. ESA: electrical spectrum analyzer. SSA: signal source analyzer. (c) Spectrum of the optomechanical frequency comb consisting of 42 harmonics from the direct ESA measurement (0–40 GHz, blue) and those reconstructed from the heterodyne detection (40–70 GHz, brown). The red line depicts the noise floor of the ESA without input.

The experimental setup for optical and mechanical characterization of the SiC optomechanical resonator is shown in Figure 1b. For optical measurements, a continuous-wave tunable laser (Toptica CTL1500, 1460–1570 nm) is used to sweep the resonances of the microdisk, with its transmission detected by a narrow-bandwidth photodetector with high voltage gains (Thorlabs PDB450C). Once an optical mode is identified, the laser detuning and power are adjusted for mechanical mode characterization and optomechanical comb generation. For example, for linear mechanical response measurements, a 12-GHz-bandwidth photodetector (Newport AD-40) is combined with a real-time spectrum analyzer (Tektronix RSA5106A, 6.2-GHz bandwidth), while the optical power is kept sufficiently low to avoid back-action. For broadband comb spectra, the detection is switched to a 33-GHz-bandwidth photodetector (Finisar XPRV2022A) and a broadband ESA (Rohde and Schwarz FSEK30, 20 Hz to 40 GHz). To access overtones beyond 40 GHz, a second tunable laser (Agilent 81680) with a frequency offset from the pump is introduced, enabling heterodyne down-conversion of higher-order tones into the measurable range of the ESA (details in Figure 3). Figure 1c presents the comb spectrum obtained directly from the ESA (0–40 GHz) together with the heterodyne-reconstructed spectrum extending up to 70 GHz, with further details discussed below.

**Figure 2:**
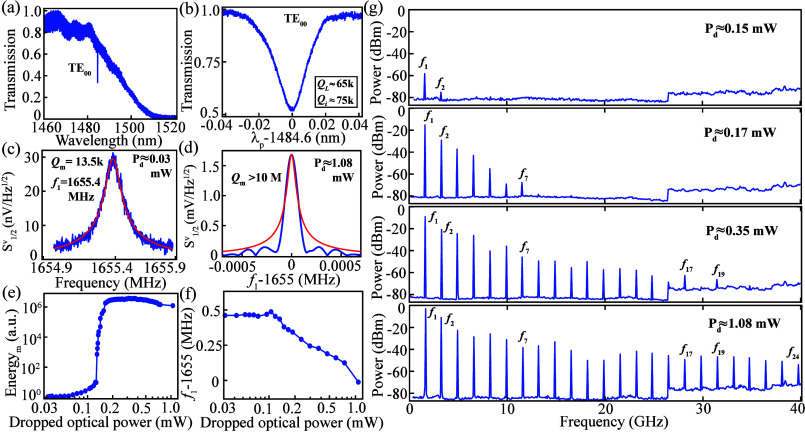
(a) Linear optical transmission of the 2.5-μm-radius SiC microdisk, in which only the fundamental transverse-electric (TE_00_) resonance around 1485 nm is observed. (b) Zoom-in scan of the TE_00_ resonance in part a. (c) Measured spectral density in voltage (blue solid line) and its Lorentzian fitting (red solid line) of the fundamental RBM of the SiC disk corresponding to a dropped optical power of 30 μW (red line represents fitting). The resolution bandwidth of the ESA is set at 20 Hz. (d) Spectral density in voltage captured within 10 ms (blue solid line) and its Lorentzian fitting (red solid line) for a dropped optical power of 1.08 mW with the resolution bandwidth set at 100 Hz. The instantaneous line width is estimated to be less than 150 Hz (corresponding *Q*_m_ above 10 million). (e and f) Mechanical energy and resonance frequency of the fundamental RBM as a function of the dropped optical power, respectively. (g) Full-range ESA spectra corresponding to various dropped optical powers.

**Figure 3:**
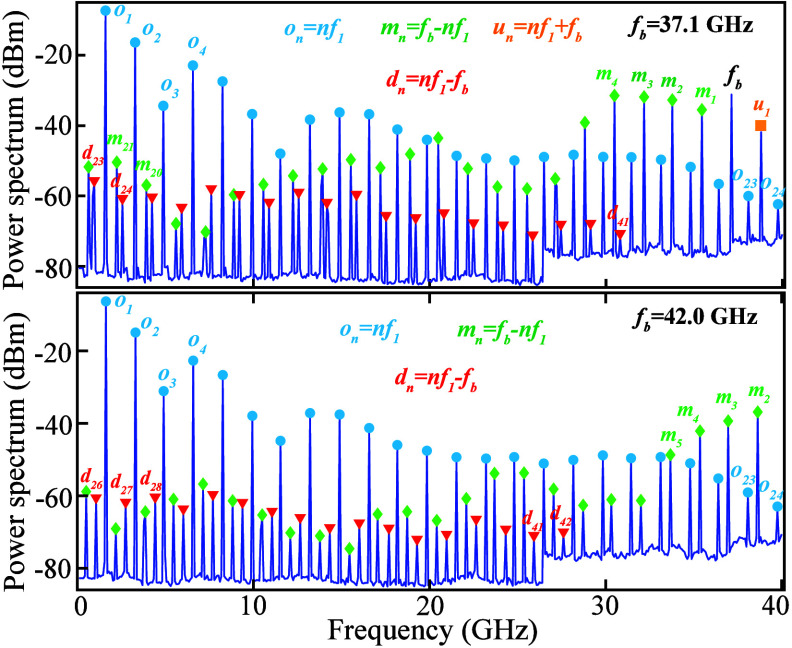
Representative ESA spectra in the heterodyne detection scheme (Figure 1b): the top and bottom panels correspond to an offset frequency (the frequency difference between the pump laser and the auxiliary laser) of 37.1 and 42.0 GHz, respectively. Here the circles, diamond, and downward triangles indicate the spectral positions of the original (*o*_*n*_), mirrored (*m*_*n*_), and down-shifted (*d*_*n*_) tones of the harmonics. The top panel includes one additional up-shifted (*u*_*n*_, square) tone in the spectrum.

In the optical characterization, light is coupled between the input/output fibers and the on-chip waveguide via grating couplers, with an approximate total insertion loss of 19 dB. Owing to the small disk size, the swept-wavelength scan typically reveals only one or two resonances in the scan range (2.5-μm radius corresponds to an optical free spectral range near 7 THz). The access waveguide geometry (500 nm width, 400 nm gap) is optimized to preferentially excite the fundamental transverse-electric (TE_00_) mode, which exhibits the highest optical *Q*. As shown in Figure 2a,b, the measured intrinsic and loaded *Q* factors of the TE_00_ resonance are 75000 and 65000, respectively, near 1485 nm. The optical power dropped into the microdisk (*P*_d_) is estimated as *P*_d_ ≈ *P*_in_(1 – *T*_o_), with *P*_in_ and *T*_o_ representing the optical power in the waveguide and the normalized optical transmission, respectively.

The fundamental radial breathing mode (RBM) of the 2.5-μm-radius microdisk is readily observed in the ESA spectrum with dropped optical power as low as 30 μW (Figure 2c). The mode exhibits a resonance frequency of 1.655 GHz and *Q*_m_ of 13,500, yielding an *f*–*Q*_m_ product of 22 THz-among the highest reported for undercut microdisks across integrated photonic platforms.^22–24^ When the dropped power exceeds 120 μW, strong phonon lasing is observed, accompanied by a rapid increase in mechanical energy (Figure 2e) and a slight frequency downshift due to the optical spring effect^23^ (Figure 2f). (Note that, although a similarly sized 3C-SiC microdisk has exhibited a 1.69-GHz mechanical mode,^22^ phonon lasing has not been observed in that system.) At the highest dropped optical power (≈1.08 mW), the RBM spectrum (Figure 1d) reveals a full width at half-maximum below 150 Hz, corresponding to *Q*_m_ > 10^7^. The measurement uncertainty is primarily due to rapid frequency fluctuations of several hundred hertz on subsecond time scales (details available in Figure 4a). To mitigate this, the spectrum in Figure 2d was acquired with a real-time ESA (Tektronix), enabling millisecond-scale scans with a relatively small resolution bandwidth (100 Hz). In Figure 2g, the full-range ESA spectra (0–40 GHz) at different optical powers portray the evolution of the optomechanical comb, with the number of observable harmonics increasing from 2 at 0.15 mW to 24 overtones at 1.08 mW. It is also noting that no subharmonics are observed in the spectrum.^27^

**Figure 4:**
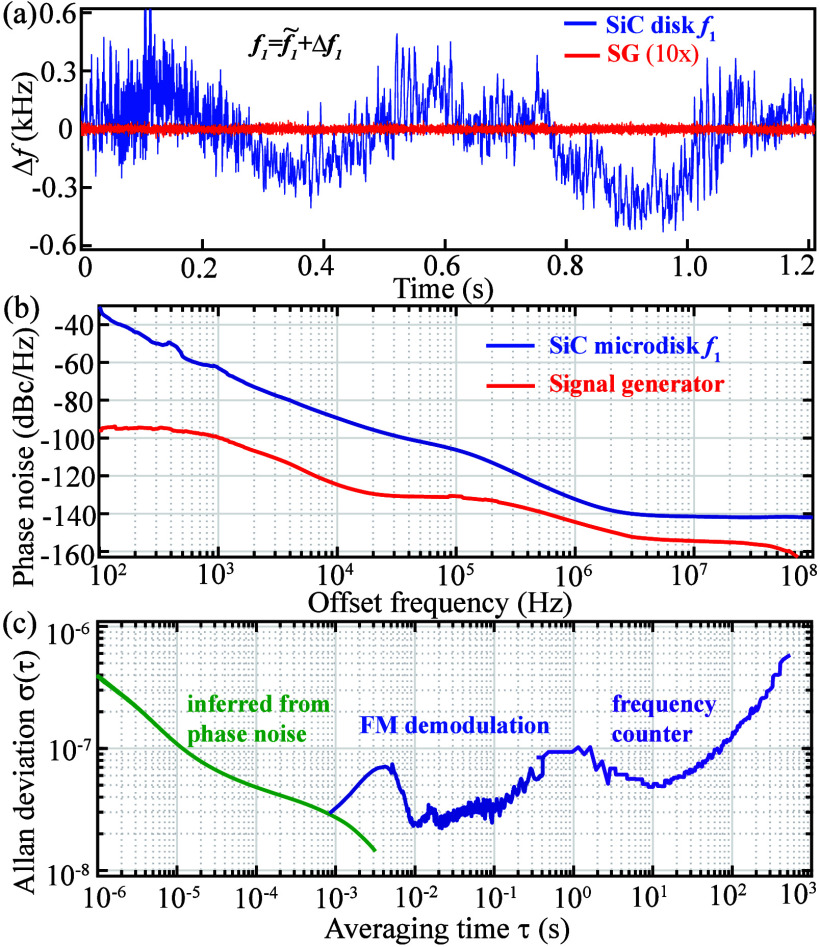
(a) Real-time frequency fluctuation (Δ*f*_1_) of the fundamental tone around its nominal value (*f̃*_1_) from the SiC microdisk (blue line) compared against that from a signal generator tuned to the same frequency (SG, red line, multiplied by a factor of 10 for visibility). (b) Single-sideband phase noise of the fundamental tone (blue solid line) and the output from a signal generator (red line). (c) Allan deviation: the green line is inferred from the phase noise in part b; the blue line is calculated based on the demodulated frequency fluctuation in part a; the purple line is acquired using the frequency counter in ESA.

Additional comb lines beyond the ESA’s 40-GHz limit are accessed by mixing the transmitted signal with an auxiliary tunable laser offset by frequency *f*_*b*_ using a 3-dB coupler (Figure 1b). This scheme resembles heterodyne detection in coherent optical communications, except that the auxiliary laser (serving as the local oscillator) is free-running and not phase-locked to the pump. As a result, the detected RF tones associated with *f*_b_ experience power fluctuations arising from time-varying phase. Their power is estimated by recording the maximum values from multiple (>10) fast ESA scans with varied offset frequencies. Two representative cases are shown in Figure 3: in the top panel (*f*_*b*_ ≈ 37.1 GHz), the spectrum contains the original harmonic tones (*o*_*n*_), the beatnote signal of the two lasers (*f*_*b*_), mirrored tones (*m*_*n*_), and down-shifted tones (*d*_*n*_). For the bottom panel, the beatnote tone *f*_*b*_ (≈42 GHz) is out of the ESA range, but similar mirrored and down-shifted tones are still observed. The overall spectrum shown in Figure 1c along with the phonon lasing threshold were compared with numerical simulations (see the Supporting Information), showing good agreement between experiment and theory.

For microwave applications, the phase noise and frequency stability of the generated comb are critical performance metrics. We first examine the fundamental tone at 1.655 GHz under maximum optical power. Using the ESA’s analog frequency demodulator, we track its real-time frequency fluctuations around the nominal value, which are on the order of 100 Hz over 10 ms time scales and can increase to ∼1 kHz within 1 s (Figure 4a). These fluctuations likely arise from multiple sources, including the free-running pump laser (whose detuning is not locked to the cavity) and variations in on-chip power due to laser and fiber-chip coupling instabilities. Despite these imperfections, the single-sideband phase noise measured with a signal source analyzer (Agilent E5052B) reaches −90, −106, and −132 dBc/Hz at 10 kHz, 100 kHz, and 1 MHz offsets, respectively (Figure 4b). Compared to a state-of-the-art signal generator (Rohde and Schwarz SMU200A) at similar frequencies, the SiC microdisk exhibits noticeably higher phase noise below 10 kHz, consistent with the frequency fluctuations observed in Figure 4a. The Allan deviation (Figure 4c) is derived using different methods depending on the averaging time scale: for averaging times below 1 ms, it is mainly estimated from the measured phase noise (offsets > 1 kHz) in Figure 4b; for averaging times between 1 ms and 0.3 s, it is calculated from the demodulated frequency fluctuations in Figure 4a; for averaging times longer than 0.3 s, direct frequency tracking is performed using the frequency counter in ESA. Overall, the oscillator achieves a frequency stability better than 10^–7^ at 1 s of averaging time, with degradation at longer times attributed to environmental temperature fluctuations.

Next, we examine the unique phase-locking property among the spectral components of our optomechanical comb. As shown in Figure 5a, frequency measurements using ESA confirm a linear relationship between the overtone frequency (*f*_*n*_) and the harmonic order (*n*), given by *f*_*n*_ = *nf̃*_1_ + Δ*f*_*n*_, where *f̃*_1_ is the nominal fundamental frequency and Δ*f*_*n*_ is the residual fitting error. Each Δ*f*_*n*_ fluctuates with time, leading to a fractional uncertainty Δ*f*_*n*_/*f*_*n*_ on the order of 10^–7^ (Figure 5b). If the overtone FC exhibits true phase locking with equal spacing, the relationship *f*_*n*_ = *nf*_1_ should hold exactly, even as *f*_1_ undergoes random fluctuations (Figure 4a). As such, the fitting errors representing real-time frequency fluctuations should scale as Δ*f*_*n*_ = *n*Δ*f*_1_. To verify this, we employ analog frequency demodulation using two synchronized ESAs: one (Tektronix RSA5106A) for the fundamental tone and the other (FSEK30) for the overtone. Representative measurements for the third and tenth harmonics, shown in Figure 5c,d, respectively, confirm strong phase locking across the comb lines. A direct time-domain measurement of the transmitted signal and its comparison against numerical modeling are also provided in the Supporting Information, which further confirms the phase-locking behavior.

**Figure 5:**
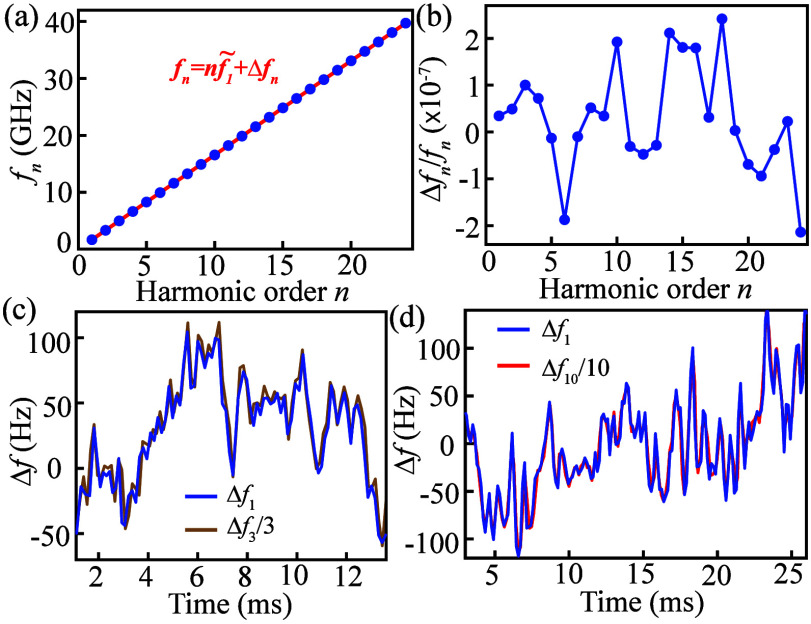
(a) Measured center frequencies of harmonic tones (blue circles) and their linear fitting (red line). (b) Fractional residual frequency errors for each harmonic from the linear fitting. (c) Simultaneously detected frequency fluctuation of the fundamental tone (blue) compared against that of the third harmonic (brown, amplitude divided by three). (d) Similar to part c but for comparison between the fundamental (blue) and the 10th harmonic (red).

In Table 1, we benchmark the performance of our SiC optomechanical frequency comb against experiments in other device platforms. As can be seen, our device stands out by simultaneously achieving a high fundamental frequency, a broad comb span, and a low phonon lasing threshold. While other platforms have reported higher harmonic counts at lower oscillation frequencies, our overtone comb uniquely enables direct access to high-frequency, phase-locked harmonics with only milliwatt-level optical power (whereas ref 17 needs 448 mW for the reported 43.5 GHz span). In addition, the measured phase noise of −132 dBc/Hz at a 1 MHz offset and frequency stability better than 10^–7^ (at 1 s integration time) are among the best reported for on-chip optomechanical oscillators, highlighting the potential of 4H-SiC for optomechanical applications.

**Table 1:** Comparison of This Work with Representative Optomechanical Frequency Combs

parameter	Mercadé et al.^16^	Luan et al.^27^	Tallur et al.^15^	de Jong et al.^20^	Hu et al.^17^	this work
material	silicon	silicon	Si_3_N_4_	Si_3_N_4_	silica	4H-SiC
optical *Q*	5k-loaded	60–150k	>300k	N.A.	113 M	65k-loaded
mechanical frequency *f*_m_	3.897 GHz	112.7 MHz	41.95 MHz	118.049 kHz	50.22 MHz	1.655 GHz
mechanical *Q*_m_	2400	480	2000	∼1 × 10^6^	5473	13500
lasing threshold	2.48 mW	127 μW	∼200 μW	N.A.	N.A.	∼120 μW
phase noise	–100 dBc/Hz at 100 kHz	–125 dBc/Hz at 10 kHz	–85 dBc/Hz at 1 kHz	N.A.	N.A.	–132 dBc/Hz at 1 MHz
comb lines	5th harmonics	59th harmonics	12 overtones	35 overtones	867 overtones	42 overtones
harmonics span	∼19.35 GHz	∼6.9 GHz	∼500 MHz	∼4.2 MHz	∼43.5 GHz	∼70 GHz

In conclusion, we demonstrated an ultracompact optomechanical frequency comb based on a 2.5-μm-radius SiC optomechanical resonator, capable of generating 42 phase-locked harmonics with a frequency spacing of 1.655 GHz under a dropped optical power of only ∼1 mW. The resulting comb spans a record 1–70 GHz and exhibits low phase noise (−132 dBc/Hz at a 1 MHz offset for the fundamental tone) and excellent frequency stability (<10^–7^ at 1s of averaging time). These performance metrics can be further improved through techniques such as laser injection locking of the SiC microdisk resonator and active feedback for compensating environmental temperature drifts. With its chip-scale form factor, broadband coverage, and phase coherence, this SiC-based optomechanical frequency comb holds strong potential for applications ranging from high-performance microwave sources in 5G/6G communications to quantum information technologies involving high-frequency microwave photons.

## Supplementary Material



## Data Availability

Data underlying the results presented in this paper are not publicly available at this time but may be obtained from the authors upon reasonable request.

## Notes

Details on the optomechanical comb modeling, phonon-lasing threshold-power estimation, and numerical simulations of 4H-SiC microdisks, along with a comparison to the experimental results (PDF)
