# Supplementary material for: Chip-Scale Optomechanical Frequency Comb with a 1–70 GHz Span
Source: Nano Lett. 2025 Dec 12;25(51):17644–9. doi: 10.1021/acs.nanolett.5c04458 (PMC12750997; doi:10.1021/acs.nanolett.5c04458)
Supplement: Supplementary file 1 [file nl5c04458_si_001.pdf]

Support Information for "Chip-scale optomechanical frequency comb with 1-70 GHz span"

Xirui Gou,[†,§] William Privratsky,[†,§] Wenhan Sun,[†] Yuncong Liu,[‡] Hamed Abiri,[¶] and Qing Li[*,†]

†*Department of Electrical and Computer Engineering, Carnegie Mellon University, Pittsburgh, PA 15213, USA*

‡*Department of Electrical and Computer Engineering, University of Florida, Gainesville, FL 32611, USA*

¶*School of Electrical and Computer Engineering, Georgia Institute of Technology, Atlanta, GA 30332 USA*

§*These authors contributed equally to this work.*

* E-mail: qingli2@andrew.cmu.edu

1. Optomechanical comb modeling

The interaction between the optical and the mechanical modes in a resonator can be described by the following two coupled mode equations that incorporate both optical and mechanical

nonlinearities:[1–3]

$$\frac{da}{dt} = \left(j(\Delta + g_{\mathrm{OM}}x) - \frac{\Gamma_t}{2}\right) a + \sqrt{\Gamma_e} s_{in}, \tag{S1}$$

$$\frac{d^2x}{dt^2} + \Gamma_m \frac{dx}{dt} + \Omega_m^2 x + c_{\mathrm{duff}} x^3 = \frac{\hbar g_{\mathrm{OM}}}{m_{\mathrm{eff}}} |a|^2, . \tag{S2}$$

where $|a|^2$ and $|s_{in}|^2$ represent the number of intracavity photons and the input photon flux, respectively; $\Delta \equiv \omega_L - \omega_0$ describes the detuning of the laser frequency (ω_L) relative to the optical resonance ω_0; x is the mechanical displacement; g_{OM} is the optomechanical coupling coefficient defined as $g_{\mathrm{OM}} \equiv -\partial\omega_0/\partial x$; Γ_t and Γ_e denote the total and external loss of the optical mode, respectively ($\Gamma_t = \Gamma_e + \Gamma_0$ with Γ_0 being the intrinsic optical loss); Γ_m, Ω_m, and m_{eff} represent the decay rate, resonance frequency, and effective mass of the mechanical mode, respectively; c_{duff} is a Duffing term introduced to account for the mechanical nonlinearity; and $\hbar$ is the Planck's constant.

For the SiC microdisk employed in the experiment, the parameters in Eqs. S1 and S2 are estimated as follows. The optomechanical coupling rate is given by: $g_{\mathrm{OM}} \approx \omega_0/R$, where $R = 2.5$ μm denotes the disk radius. The total, external, and intrinsic optical decay rates are expressed as $\Gamma_t = \omega_0/Q_L$, $\Gamma_e = \omega_0/Q_c$, and $\Gamma_0 = \omega_0/Q_0$, with Q_L, Q_c, Q_0 representing the loaded, coupling, and intrinsic optical quality factors, respectively. The input field amplitude s_{in} satisfies $|s_{in}|^2 = P_{in}/(\hbar\omega_0)$, where P_{in} is the on-chip optical power in the bus waveguide. The mechanical frequency is $\Omega_m/(2\pi) \approx 1.655$ GHz, and the mechanical damping rate is $\Gamma_m = \omega_m/Q_m$, where Q_m is the mechanical Q in the linear regime ($Q_m \approx 13,500$).

The effective mass m_{eff} generally differs from the total mass of the SiC microdisk and is defined as:[4]

$$m_{\mathrm{eff}} \equiv \frac{2U}{x_{max}^2 \Omega_m^2}, \tag{S3}$$

where U and x_{max} denote the total mechanical energy and the maximum displacement amplitude, respectively. For the 2.5-μm-radius SiC microdisk, m_{eff} is estimated to be 60% of the total mass based the finite element method.[5]

Finally, the Duffing coefficient c_{duff} can be extracted by fitting the experimental response to numerical simulations. However, accurate estimation requires extensive data due to the complex dynamics of the coupled nonlinear system and uncertainties in the parameters used for modeling. For simplicity, we have set $c_{\text{duff}} = 0$ in the numerical simulations performed in this document.

2. Phonon lasing threshold power estimation

The phonon lasing threshold can be deduced from Eqs. S1 and S2 using small-signal analysis:[1,6]

$$P_d = \frac{m_{\text{eff}}\omega_0}{2g_{\text{OM}}^2}\frac{\Gamma_0\Gamma_m}{\Gamma_t\Delta}\left[(\Delta-\Omega_m)^2+\left(\frac{\Gamma_t}{2}\right)^2\right]\left[(\Delta+\Omega_m)^2+\left(\frac{\Gamma_t}{2}\right)^2\right]. \tag{S4}$$

A list of the physical parameters is provided in Table S1, where we have included the vacuum optomechanical coupling rate g_0 which is defined as $g_0 \equiv g_{\text{OM}}\sqrt{\hbar/(2m_{\text{eff}}\Omega_m)}$ and the single-photon cooperativity C_0 which is defined as $C_0 \equiv 4g_0^2/(\Gamma_t\Gamma_m)$. In addition, the effective cooperativity C_{eff} corresponding to a dropped optical power of 1 mW is also calculated, using the computed intracavity photon number of 2.6×10^5 (see Fig. S1a, $C_{\text{eff}} = N_{ph}C_0$).

Table S1: Summary of key parameters used in the power threshold calculation and comb modeling.

**Domain**	**Parameters**	**Description**	**Values**
Optical	$\omega_0/2\pi$	Optical frequency	202.1 THz
	$Q_L \mid Q_i \mid Q_c$	Optical Q factors	65k | 75k | 488k
	$\Gamma_t \mid \Gamma_0$	Loaded | intrinsic photon decay rate	19.5 | 16.9 ns^{-1}
	$\Delta/2\pi$	Laser-cavity detuning	1.24 GHz
Mechanical	m_{eff}	Effective mass	19.1 pg
	$\Omega_m/2\pi$	Mechanical frequency	1.655 GHz
	Q_m	Mechanical quality factor	13,500
	Γ_m	Mechanical energy decay rate	0.77 μs^{-1}
Optomechanical	$g_{\text{OM}}/2\pi$	Optomechanical coupling coefficient	80.8 GHz/nm
	$g_0/2\pi$	Vacuum OM coupling rate	41.6 kHz
	C_0	Single-photon cooperativity	1.8×10^{-5}
	C_{eff}	Effective cooperativity at $P_d = 1$ mW	4.7

By substituting the parameters into Eq. S4, we obtain a phonon lasing threshold of approximately 0.17 mW, which is slightly higher than the experimentally observed value of 0.12 mW (see Fig. 2e in the main text). This discrepancy is primarily attributed to uncertainties in several parameters, particularly in g_{OM}, which in the current estimation accounts only for the moving boundary contribution while neglecting other effects such as the photoelastic coupling.

3. Numerical simulations and comparison with experiment

Numerical simulations based on the Eqs. S1 and S2 have also been carried out corresponding to parameters listed in Table S1. For example, at the maximum input power ($P_d \approx 1.08$ mW), we plot the results in Fig. S1 for the intracavity photon number and the displacement, both of which evolve into stable oscillations from the initial zero condition. The estimated displacement amplitude is about 40 pm after the oscillation stabilizes.

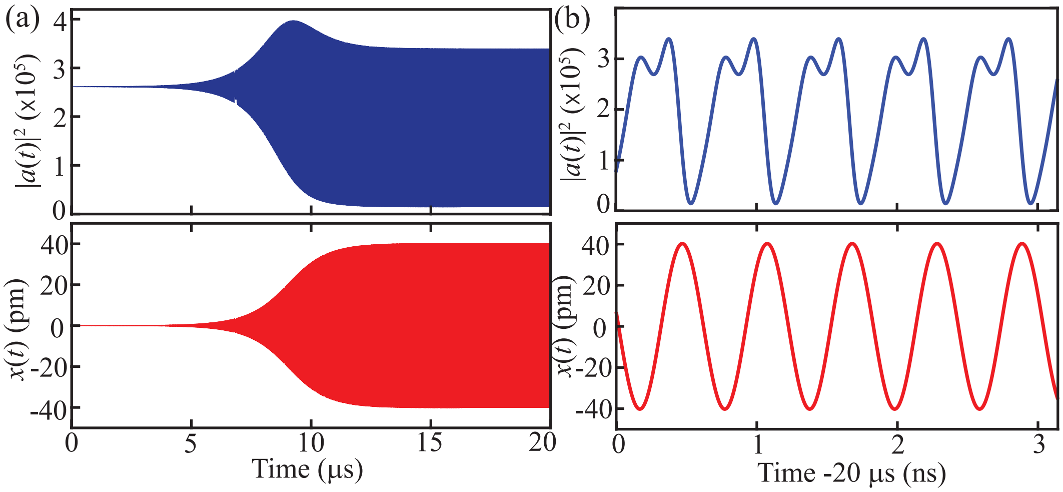

Figure S1: (a) Simulated intracavity photon number ($|a(t)|^2$) and the displacement as a function of the simulation time. (b) a zoom-in plot of (a) to reveal the cycles.

The simulation results also enable a direct comparison with the experimental data presented in the main text. In the experiment, the detected signal corresponds to the transmission from the bus waveguide, which can be computed as $s_t = s_{in} - \sqrt{\kappa_e}a$. The top panel of

Fig. S2a shows the simulated transmission in the time domain under stable oscillation. For verification, the RF output from the 12-GHz photodetector was measured using a 6-GHz-bandwidth oscilloscope, and the corresponding trace is plotted in the lower panel of Fig. S2a. As shown, the simulation and experimental results exhibit good agreement.

In the frequency domain, the numerical simulation (Fig. S1a) was extended to a duration of 200 μs to suppress spurious noise in the Fourier transform of the time-domain signal (top panel of Fig. S2b). The resulting spectrum closely resembles the experimental observation, with minor discrepancies likely arising from nonuniform frequency responses in the photodetector, RF cables, and other components in the measurement chain.

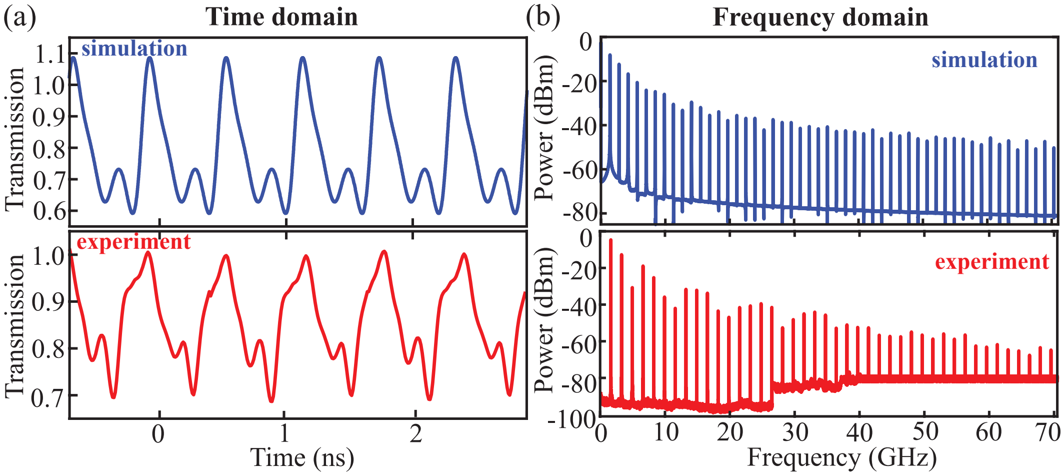

Figure S2: (a) and (b): comparison between simulation and experiment in the time and frequency domain for a dropped optical power of 1.08 mW, respectively.

References

(1) Miri, M.-A.; D'Aguanno, G.; Alù, A. Optomechanical frequency combs. *New Journal of Physics* **2018**, *20*, 043013, Publisher: IOP Publishing.

(2) Mercadé, L.; Martín, L. L.; Griol, A.; Navarro-Urrios, D.; Martínez, A. Microwave oscillator and frequency comb in a silicon optomechanical cavity with a full phononic bandgap. *Nanophotonics* **2020**, *9*, 3535–3544.

(3) de Jong, M. H. J.; Ganesan, A.; Cupertino, A.; Gröblacher, S.; Norte, R. A. Mechanical overtone frequency combs. *Nature Communications* **2023**, *14*, 1458.

(4) Hossein-Zadeh, M.; Rokhsari, H.; Hajimiri, A.; Vahala, K. J. Characterization of a radiation-pressure-driven micromechanical oscillator. *Physical Review A* **2006**, *74*, 023813, Publisher: American Physical Society.

(5) Liu, Y.; Sun, W.; Abiri, H.; Feng, P. X.-L.; Li, Q. Ultracompact 4H-silicon Carbide Optomechanical Resonator with $F_m \cdot Q_m$ Exceeding 10^{13} Hz. *Photonics Research* **2025**, *13*, 2531–2538.

(6) Jiang, W. C.; Lu, X.; Zhang, J.; Lin, Q. High-frequency silicon optomechanical oscillator with an ultralow threshold. *Optics Express* **2012**, *20*, 15991–15996.
